# Alcohol Intake and Blood Pressure: A Systematic Review Implementing a Mendelian Randomization Approach

**DOI:** 10.1371/journal.pmed.0050052

**Published:** 2008-03-04

**Authors:** Lina Chen, George Davey Smith, Roger M Harbord, Sarah J Lewis

**Affiliations:** 1 Department of Social Medicine, University of Bristol, Bristol, United Kingdom; 2 Medical Research Council Centre for Causal Analyses in Translational Epidemiology, University of Bristol, Bristol, United Kingdom; Imperial College London, United Kingdom

## Abstract

**Background:**

Alcohol has been reported to be a common and modifiable risk factor for hypertension. However, observational studies are subject to confounding by other behavioural and sociodemographic factors, while clinical trials are difficult to implement and have limited follow-up time. Mendelian randomization can provide robust evidence on the nature of this association by use of a common polymorphism in *aldehyde dehydrogenase 2* (*ALDH2*) as a surrogate for measuring alcohol consumption. *ALDH2* encodes a major enzyme involved in alcohol metabolism. Individuals homozygous for the null variant (*2*2) experience adverse symptoms when drinking alcohol and consequently drink considerably less alcohol than wild-type homozygotes (*1*1) or heterozygotes. We hypothesise that this polymorphism may influence the risk of hypertension by affecting alcohol drinking behaviour.

**Methods and Findings:**

We carried out fixed effect meta-analyses of the *ALDH2* genotype with blood pressure (five studies, *n* = 7,658) and hypertension (three studies, *n* = 4,219) using studies identified via systematic review. In males, we obtained an overall odds ratio of 2.42 (95% confidence interval [CI] 1.66–3.55, *p* = 4.8 × 10^−6^) for hypertension comparing *1*1 with *2*2 homozygotes and an odds ratio of 1.72 (95% CI 1.17–2.52, *p* = 0.006) comparing heterozygotes (surrogate for moderate drinkers) with *2*2 homozygotes. Systolic blood pressure was 7.44 mmHg (95% CI 5.39–9.49, *p* = 1.1 × 10^−12^) greater among *1*1 than among *2*2 homozygotes, and 4.24 mmHg (95% CI 2.18–6.31, *p* = 0.00005) greater among heterozygotes than among *2*2 homozygotes.

**Conclusions:**

These findings support the hypothesis that alcohol intake has a marked effect on blood pressure and the risk of hypertension.

## Abstract 2

Using a mendelian randomization approach Sarah Lewis and colleagues find strong support for the hypothesis that alcohol intake has a marked effect on blood pressure and the risk of hypertension.

## Editors' Summary

**Background.:**

High blood pressure (hypertension) is a common medical condition that affects nearly a third of US and UK adults. Hypertension has no symptoms but can lead to heart attacks or strokes. It is diagnosed by measuring blood pressure—the force that blood moving around the body exerts on the inside of large blood vessels. Blood pressure is highest when the heart is pumping out blood (systolic pressure) and lowest when it is filling up with blood (diastolic pressure). Normal blood pressure is defined as a systolic pressure of less than 130 millimeters of mercury (mmHg) and a diastolic pressure of less than 85 mmHg (a blood pressure of 130/85). A reading of more than 140/90 indicates hypertension. Many factors affect blood pressure, but overweight people and individuals who eat too much salty or fatty foods are at high risk of developing hypertension. Mild hypertension can often be corrected by lifestyle changes, but many people also take antihypertensive drugs to reduce their blood pressure.

**Why Was This Study Done?:**

Another modifiable lifestyle factor thought to affect blood pressure is alcohol intake. Observational studies that ask people about their drinking habits and measure their blood pressure suggest that alcohol intake correlates with blood pressure, but they cannot prove a causal link because of “confounding”—other risk factors associated with alcohol drinking, such as diet, might also affect the study participant's blood pressures. A trial that randomly assigns people to different alcohol intakes could provide this proof of causality, but such a trial is impractical. In this study, therefore, the researchers have used “Mendelian randomization” to investigate whether alcohol intake affects blood pressure. An inactive variant of aldehyde dehydrogenase 2 (ALDH2; the enzyme that removes alcohol from the body) has been identified. People who inherit the variant form of this gene from both parents have an ALDH2 *2*2 genotype (genetic makeup) and become flushed and nauseated after drinking. Consequently, they drink less than people with a *1*2 genotype and much less than those with a *1*1 genotype. Because inheritance of these genetic variants does not affect lifestyle factors other than alcohol intake, an association between *ALDH2* genotypes and blood pressure would indicate that alcohol intake has an effect on blood pressure without any confounding.

**What Did the Researchers Do and Find?:**

The researchers identified ten published studies (mainly done in Japan where the *ALDH2* gene variant is common) on associations between *ALDH2* genotype and blood pressure or hypertension using a detailed search protocol (a “systematic review”). A meta-analysis (a statistical method for combining the results of independent studies) of the studies that had investigated the association between *ALDH2* genotype and hypertension showed that men with the *1*1 genotype (highest alcohol intake) and those with the *1*2 genotype (intermediate alcohol intake) were 2.42 and 1.72 times more likely, respectively, to have hypertension than those with the *2*2 genotype (lowest alcohol intake). There was no association between *ALDH2* genotype and hypertension among the women in these studies because they drank very little. Systolic and diastolic blood pressures showed a similar relationship to *ALDH2* genotype in a second meta-analysis of relevant studies. Finally, the researchers estimated that for men the lifetime effect of drinking 1 g of alcohol a day (one unit of alcohol contains 8 g of alcohol in the UK and 14 g in the US; recommended daily limits in these countries are 3–4 and 1–2 units, respectively) would be an increase in systolic blood pressure of 0.24 mmHg.

**What Do These Findings Mean?:**

These findings support the suggestion that alcohol has a marked effect on blood pressure and hypertension. Consequently, some cases of hypertension could be prevented by encouraging people to reduce their daily alcohol intake. Although the Mendelian randomization approach avoids most of the confounding intrinsic to observational studies, it is possible that a gene near *ALDH2* that has no effect on alcohol intake affects blood pressure, since genes are often inherited in blocks. Alternatively, ALDH2 could affect blood pressure independent of alcohol intake. The possibility that ALDH2 could effect blood pressure independently of alcohol is intake made unlikely by the fact that no effect of genotype on blood pressure is seen among women who drink very little. Additional large-scale studies are needed to address these possibilities, to confirm the current finding in more people, and to improve the estimates of the effect that alcohol intake has on blood pressure.

**Additional Information.:**

Please access these Web sites via the online version of this summary at http://dx.doi.org/10.1371/journal.pmed.0050052.

- The MedlinePlus encyclopedia has a page on hypertension  (in English and Spanish)
- The American Heart Association provides information for patients and health professionals about hypertension
- The UK Blood Pressure Association provides information for patients and health professionals on all aspects of hypertension, including information about alcohol affects blood pressure
- The Explore@Bristol science center (a UK charity) provides an alcohol unit calculator and information on the effects of alcohol
- The International Center for Alcohol Policies provides drinking guidelines for countries around the world

## Introduction

Observational epidemiological studies have generally reported that blood pressure is lower among individuals with a moderate alcohol intake than in nondrinkers, but that heavy alcohol intake is associated with an increase in blood pressure [1–4]. However, associations in observational studies of alcohol intake and blood pressure may be heavily confounded by factors such as diet, smoking, exercise levels, and socioeconomic position. Conversely, the detection of high blood pressure may lead to efforts to reduce alcohol intake, which will attenuate any positive association. In addition, reporting of alcohol is likely to be subject to considerable error, and this error may be differential—e.g., people who have been advised to reduce alcohol intake for medical reasons may under-report alcohol intake.

Randomized controlled trials in which individuals are randomized to drink different amounts of alcohol are problematic for ethical reasons and in terms of compliance, so trials have generally randomized individuals who drink at least moderate amounts of alcohol to receive interventions aimed at reducing drinking. It is difficult to know how successful such interventions are, and how taking part in a trial aimed at reducing alcohol intake influences reporting of (rather than actual) drinking behaviour. Furthermore, follow-up is generally short-term, whereas alcohol intake may have long-term effects on blood pressure.

Mendelian randomization can provide robust evidence on such associations [5]. Alcohol is initially metabolised to an intermediate compound, acetaldehyde, which is further metabolised and then eliminated from the body. The major enzyme responsible for the elimination of acetaldehyde is alcohol dehydrogenase 2 (ALDH2) [6]. In some populations, the *ALDH2* gene is polymorphic and an individual's genotype at this locus influences blood acetaldehyde concentrations after drinking [7]. A single point mutation in *ALDH2* has resulted in the ALDH2*2 allele, which is common in some Asian populations. The resultant protein has an amino acid substitution from glutamic acid (glutamate) to lysine at residue 487, and an inactive subunit. This substitution leads to an inability to metabolize acetaldehyde and causes an accumulation of acetaldehyde after alcohol intake [7]. Individuals who are homozygous for the ALDH2*2 allele have 18 times higher, and heterozygotes have five times higher, peak blood acetaldehyde levels compared with *1*1 homozygotes (wild type) [8]. ALDH2*2*2 homozygotes are characterized by a facial flushing response after consumption of alcohol coupled with nausea, drowsiness, headache, and other unpleasant symptoms that normally prevent them from heavy drinking [8]. Heterozygotes have a limited ability to metabolize acetaldehyde, but exhibit a less severe reaction than that seen among ALDH2*2*2 homozygotes. Due to the above adverse symptoms when drinking alcohol, *2*2 homozygotes drink considerably less alcohol than people homozygous for the wild-type allele (*1), with heterozygotes drinking intermediate amounts [9]. The genetic variant is not, however, associated with factors that would confound the relationship of alcohol consumption and blood pressure (such as smoking) nor, of course, would germline genetic variants be influenced by blood pressure level. Thus genotype can define groups with considerably different levels of lifetime average alcohol intake but with no difference in confounding factors and without the influence of reverse causation or differential reporting bias. Analysing individuals by genotype is akin to a randomised controlled trial of different levels of alcohol intake. This approach has been used to provide proof of concept—the genotype related to higher alcohol intake is also associated with the predicted increase in oesophageal cancer risk, a disease generally considered to be alcohol related [9].

We have used a Mendelian randomization approach [5] to establish the extent to which alcohol intake results in changes in blood pressure, using *ALDH2* genotype as an instrument for indexing alcohol intake at a group level, producing groups with relatively high (*1*1), moderate (*1*2), and very low alcohol consumption (*2*2). We undertook a systematic review and carried out meta-analyses of studies examining the association of *ALDH2* with risk of hypertension and/or level of blood pressure.

## Methods

### Search Strategy

**Figure 1:**
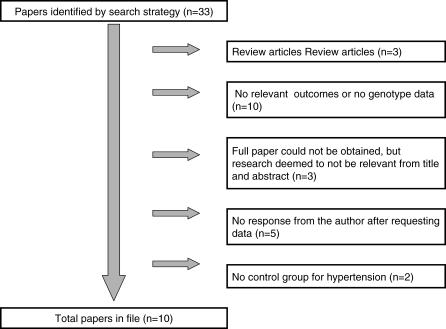
Flow Diagram Showing Reasons for Exclusion and Number of Papers Excluded

Papers included in the systematic review were retrieved from Medline (http://www.ncbi.nlm.nih.gov/PubMed) and ISI web of Knowledge (http://wok.mimas.ac.uk/) using the search terms: “Aldehyde dehydrogenase 2” and “ALDH2” in combination with “Hypertension”, “Blood pressure”, “Cardiovascular diseases”, “Coronary disease”, “Heart disease”, “Coronary artery disease”. The bibliographies of retrieved articles were also scanned for relevant publications. We searched for all papers published before October 2006. Screening of studies for inclusion was performed by two researchers (LC, SJL) independently of each other to increase objectivity. We also searched for all studies of the *ALDH2* genotype regardless of outcome for use in separate analyses, and we scanned the abstracts of these studies to determine whether they may include any of the outcomes we were interested in. If we were uncertain we scanned the paper and then contacted the authors. There was no exclusion on the basis of language. For multiple publications based on the same study, the latest publication was included. For inclusion we required, as a minimum, data on systolic or diastolic blood pressure by genotype or prevalence of hypertension by genotype. Where insufficient data for inclusion were available in the paper we contacted authors directly, and if sufficient information could still not be obtained the study was excluded. A flow diagram showing the number of studies found in our search strategy, and reasons for exclusion is given in Figure 1.

### Data Extraction

We followed a standard protocol for data extraction. For each study, the following data were recorded (where available): first author's name, year of publication, country and city in which the study was performed, name of the study, study design, number and source of participants, sex, method of assessment of drinking status, categories of alcohol drinking, mean alcohol drinking and standard deviation by *ALDH2* genotype, distribution of potential confounding factors by genotype with *p*-values, distribution of genotypes among hypertensive and nonhypertensive participants, mean blood pressure and standard deviation by *ALDH2* genotype, and reported effect estimates (i.e., relative risks, odds ratios, or mean difference of blood pressure level) and 95% confidence intervals (CIs) for *ALDH2* genotype and hypertension risk. Data extraction was carried out by two researchers (LC, SJL) independently of each other.

### Statistical Analysis

Data on alcohol intake were converted to a uniform measure of grams per day for comparison across all studies with 1 unit = 10 ml = 7.9 g [10].

We calculated the unadjusted mean difference in blood pressure or the odds ratio of hypertension by comparing *1*2 and *1*1 to *2*2 genotypes. In the analyses of hypertension, fixed-effects meta-analysis was used to calculate summary odds ratio estimates, and in the analyses of diastolic and systolic blood pressure, mean differences between genotype groups were obtained. We carried out fixed-effects meta-analyses separately for men and women, excluding two very small studies (Nishimura [11], *n* = 36, and Mackenzie [12], *n* = 28) that did not provide data separately by sex, and two further small studies [13,14] for which we were unable to obtain estimates of blood pressure separately for *1*2 and *2*2 genotypes. All statistical analyses were carried out with the use of Stata statistical software (version 9.2; Stata Corporation). All statistical tests were two-sided. We assessed evidence of small-study effects (which may include publication bias) for studies of *ALDH2* polymorphism and hypertension risk, and for *ALDH2* and blood pressure by computing both the Egger [15] and Begg [16] tests.

For the studies that reported both blood pressure and mean alcohol intake by genotype, we calculated estimates of the causal effect of alcohol on blood pressure, using the method of instrumental variables with genotype as the instrument assuming the effect is linear. In order to use standard instrumental variable estimation methods (‘two-stage least squares') [17] the corr2data command in Stata was used to construct an artificial dataset matching the reported number of participants with each genotype, and the means and standard deviations of alcohol intake and blood pressure within each genotype. One study reported the mean but not the standard deviation of alcohol intake by genotype [18], so it was estimated from a histogram. As the correlation between alcohol intake and blood pressure within each genotype was not available, this correlation was assumed to be zero, which is slightly conservative, as the standard error of the estimated effect of alcohol on blood pressure reduces slightly as this correlation increases. As a sensitivity analysis we repeated the analysis assuming a correlation of 0.2 in all genotypes and all studies. Instrument strength was assessed using the first-stage *F*-statistic; values considerably above 10 are generally taken to show sufficient instrument strength to ensure the validity of two-stage least-squares [17]. The resulting estimates were then meta-analysed using the same methods as above.

## Results

Table 1 provides details of the studies identified in our systematic review and included in our meta-analysis. The systematic review identified ten articles reporting on associations between *ALDH2* and hypertension or blood pressure, the majority of which were cross sectional studies; five of these studies focused on male populations only (Table 1). All studies were population-based and the majority of participants were Japanese except one very small study carried out in the UK [12]. Tables 2 and 3 show associations between genotype and alcohol intake, and between genotype and potential confounders, with *p*-values taken from the original papers. All studies showed substantial differences in alcohol intake by genotype among men; among women alcohol intake was very low, but studies showed trends in the same direction as men. Whilst it is not possible to combine data on alcohol intake by genotype across all studies due to inconsistent and incomplete reporting of data, the two largest studies [18,19] that contributed most of the weight to the meta-analysis showed alcohol intake levels among men were around 20–30 g/d in *1*1 homozygotes, 10–15 g/d in heterozygotes and 0–2 g/d in *2*2 homozygotes. Table 3 shows that *ALDH2* genotype was not consistently or strongly associated with sex, smoking, or physical activity, although there were minor differences in BMI in men but not women in the two largest studies [18,19].

**Table 1:**
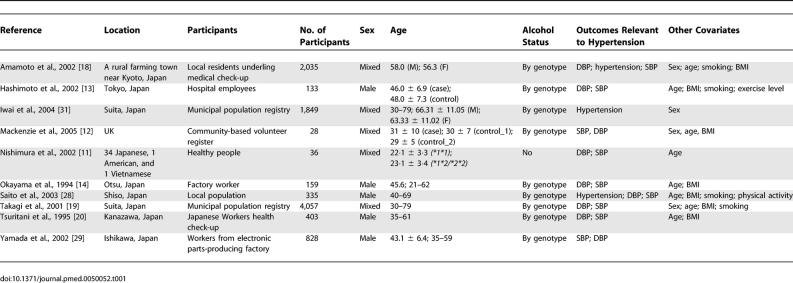
Outline of Studies Included in the Meta-analysis

**Table 2:**
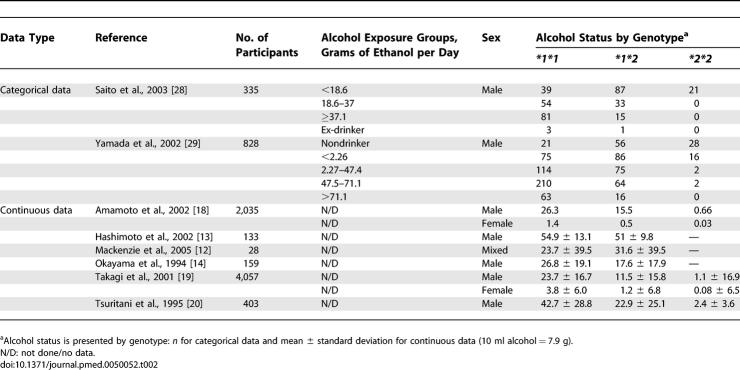
The Distribution of Alcohol Intake by Genotype in the Studies Included in This Meta-analysis

**Table 3:**
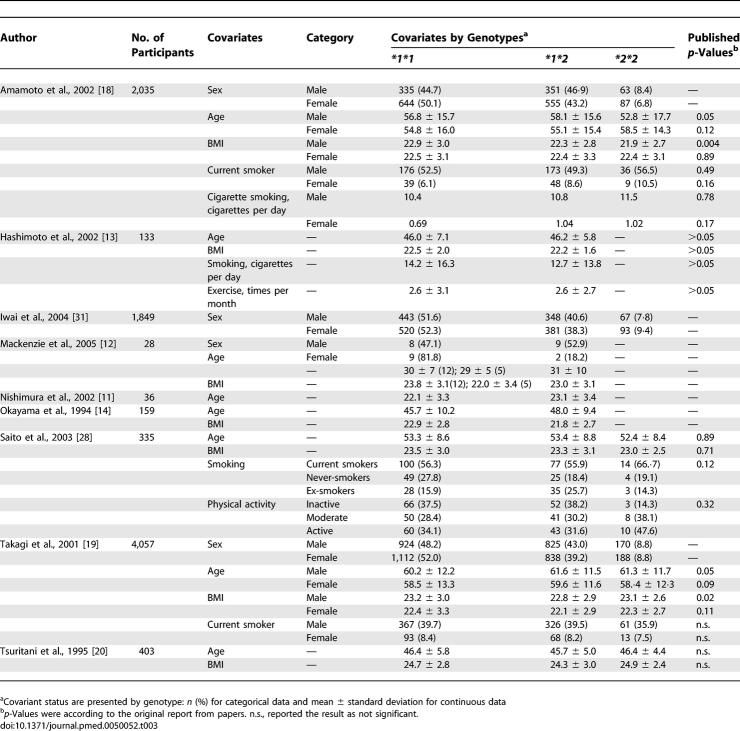
Distribution of Potential Confounding Factors by Genotype among Studies Included in the Meta-analysis

**Figure 2:**
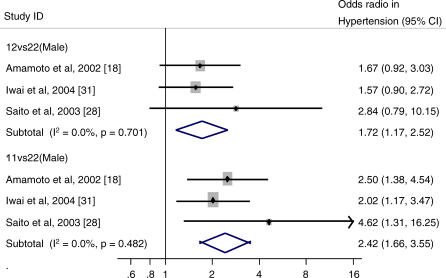
Forest Plot of Studies of *ALDH2* Genotype and Hypertension

Meta-analysis with hypertension as the outcome included 4,219 participants from three publications. In males, the pooled odds ratio was 2.42 (95% CI 1.66–3.55, *p* = 4.8 × 10^−6^) for *1*1 versus *2*2 and 1.72 (95% CI 1.17–2.52, *p* = 0.006) (Figure 2). No association between *ALDH2* and hypertension was found in females, for whom drinking levels in any genotype group were low.

**Figure 3:**
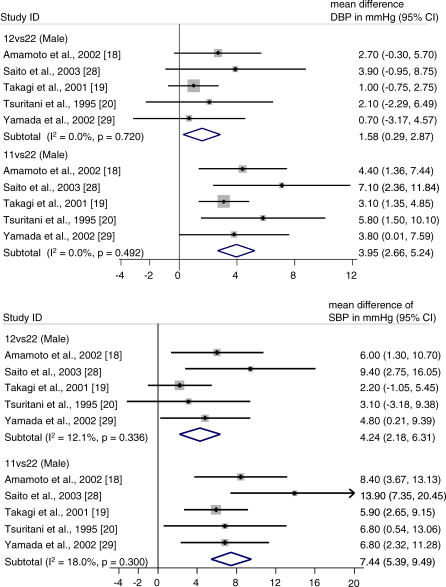
Forest Plot of Studies of *ALDH2* Genotype and Blood Pressure

Meta-analyses of diastolic blood pressure (DBP) and systolic blood pressure (SBP) were performed on 7,658 participants from five publications. Results were similar to the meta-analysis of hypertension, with mean differences in blood pressure by *ALDH2* genotype being observed in males only (Figure 3). Mean DBP in males with the *1*1 genotype was 3.95 mmHg higher than among *2*2 homozygotes (95% CI 2.66–5.24, *p* = 1.78 × 10^−9^), while among heterozygotes mean DBP was 1.58 mmHg higher than among *2*2 homozygotes (95% CI 0.29–2.87, *p* = 0.016). A similar result was obtained for SBP, with a mean difference of 7.44 mmHg (95% CI 5.39–9.49, *p* = 1.1 × 10^−12^) between *1*1 and *2*2 homozygotes, and a mean difference of 4.24 mmHg (95% CI 2.18–6.31, *p* = 0.00005) between heterozygotes and *2*2 homozygotes.

There was no strong evidence of heterogeneity between the studies. In the meta-analysis of hypertension comparing *1*2 heterozygotes and *1*1 homozygotes there was some evidence that effect sizes were greater in smaller studies (Begg's test *p* = 0.12; Egger's test *p* = 0.05). A small-study effect was also detected in the analysis of male DBP differences between *ALDH2* genotypes (DBP: *1*1 versus *2*2 Begg's test *p* = 0.05; Egger's test *p* = 0.04), no other associations showed evidence of small study bias. Sensitivity analysis including only studies with more than 500 individuals gave the following results: hypertension *1*1 versus *2*2 OR = 2.23 (95% CI 1.49–3.33; *p* = 0.00009), *1*2 versus *2*2 OR= 1.62 (95% CI 1.08–2.42; *p* = 0.02); DBP *1*1 versus *2*2 mean difference = 3.48 mmHg (95% CI 2.07–4.88; *p* = 1.3 × 10^−6^), *1*2 versus *2*2 mean difference = 1.33 mmHg (95% CI −0.07 to 2.74; *p* = 0.063) and SBP *1*1 versus *2*2 mean difference = 6.73 mmHg (95% CI 4.43–9.03; *p* = 9.7 × 10^−9^), *1*2 versus *2*2 mean difference = 3.78 mmHg (95% CI 1.47–6.09; *p* = 0.01).

**Figure 4:**
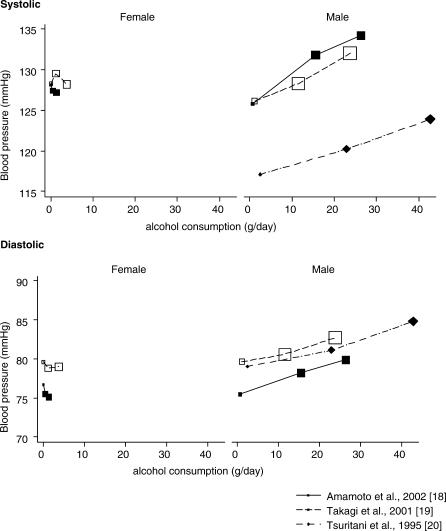
Mean Blood Pressure in Each Genotype Plotted against Mean Alcohol Consumption in Each Genotype in the Three Studies That Gave Data for Both Relationships Top graph: systolic blood pressure. Bottom graph: diastolic blood pressure. Larger plotting symbols indicate a greater number of individuals.

**Figure 5:**
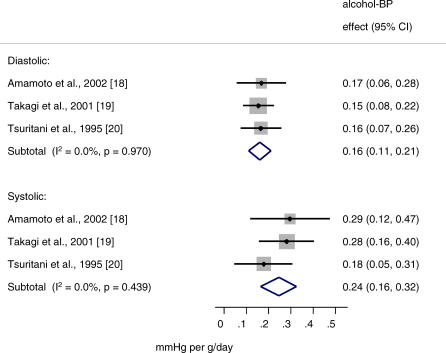
Forest Plot of Instrumental Variable Estimates of Alcohol–Blood Pressure Effect in Males Obtained Using Genotype as an Instrument for Alcohol

Figure 4 shows the relationship between the genotype means of alcohol consumption and blood pressure in the three studies that reported data for both. We see that alcohol intake level was low among females and that neither alcohol intake nor blood pressure varied noticeably by genotype. In males there was clear variation in both and the relationship between the two appears to be close to linear. The study [20] that reported greater variation in alcohol intake by genotype than the other two studies also reported greater variation in blood pressure, so that the slope of the predicted alcohol–blood pressure effect appeared similar in all studies. We examined this relationship more formally using instrumental variables. The first-stage *F*-statistics ranged from 42 [20] to 1,962 [19], indicating that genotype is a strong instrument for estimating alcohol consumption and all three studies are large enough to ensure the validity of the instrumental variables method used. The resulting estimates of the effect of alcohol on blood pressure in males are shown in the forest plot in Figure 5. No between-study heterogeneity was detected in the effect for either systolic or diastolic blood pressure, although power to detect heterogeneity was limited by the small number of studies. The meta-analytic summary estimate of the effect of alcohol intake on DBP was 0.16 (95% CI 0.11–0.21) mmHg per g/d, while the summary estimate of the effect on SBP was slightly larger at 0.24 (95% CI 0.16–0.32) mmHg per g/d. The results were essentially unchanged in the sensitivity analysis that assumed a correlation between alcohol and blood pressure within each genotype of 0.2 rather than zero: the instrumental variable estimates for each study were unchanged, while their standard errors reduced by between 1% and 7%.

## Discussion

We carried out a meta-analysis of the association between the *ALDH2* genotype and blood pressure, in which we used *ALDH2* genotype as a marker of differing alcohol intake levels. We found that individuals with the ALDH2 *1*1 genotype, with average alcohol intakes of 20–30 g/d, had strikingly higher systolic and diastolic blood pressure than those with the ALDH*2*2 genotype, who tend to drink only small amounts of alcohol. In addition, the risk of hypertension among *1*1 homozygotes was around 2.5-fold above that among *2*2 homozgotes. Even heterozygotes who tended to be quite modest drinkers had elevated blood pressure and a 70% increased risk of hypertension compared with individuals with the *2*2 genotype. Exploratory analyses using an instrumental variable approach in which the association between genotype and alcohol intake and the association between genotype and blood pressure was triangulated to give an estimate of the effect of alcohol on blood pressure, we obtained estimates of a 0.24 mmHg increase in SBP per gram of alcohol per day over the course of a lifetime (because genotype influences on alcohol intake will represent differences that generally persist throughout adult life).

It is difficult to compare these effects with those seen in randomized controlled trials, because very few trials have managed to include a control group drinking little or no alcohol, and randomized controlled trials look at the effect of changes in alcohol consumption over a short time period, whereas genetic variants will influence alcohol intake over a lifetime. One very small (*n* = 10) randomized crossover trial published in 1986 found that among healthy male volunteers, 7 d abstaining from alcohol was associated with an decrease in SBP of 3.00 mmHg relative to when drinking 60 g/d [21], although it should be emphasised that this very small study examined changes over a short time period.

Studies that have monitored blood pressure after administration of alcohol have found biphasic effects of alcohol, with acute vasodilation effects immediately after ingestion followed by increases in blood pressure in a dose-dependent manner [22]. It is not clear how much of this increase is a short-term transient effect or how this translates into longer-term effects on blood pressure levels [22]. The recent suggestion that there is no good evidence that reducing alcohol intake will reduce blood pressure reflects this uncertainty [23].

Two studies [13,18] included in this review found that body mass index (BMI) is slightly higher among *1*1 individuals than among individuals with other genotypes. This effect was seen among men, but not among women. As alcohol is an energy-dense macronutrient and has an appetite-enhancing effect [24], it is likely that the increase in BMI among individuals with the *1*1 genotype is due to their increased alcohol consumption. In support of this a positive association between BMI and alcohol intake has been observed among men in epidemiological studies [25]. A cohort study carried out in a Toyama, Japan among factory employees with an age range of 35 to 59 years found that for each 1 unit increase in BMI, SBP increased by 0.86 mmHg and DBP increased by 0.69 mmHg [26]. BMI was on average 1 unit higher among individuals with the *1*1 genotype than among those with the *2*2 genotype in the study by Amamoto et al. [18] included in this review. The predicted blood pressure effect of genotype differences in BMI equates to 11.6% of the observed difference in SBP and 16.3% of the observed difference in DBP by genotype. Therefore a small proportion of the genotype differences in blood pressure in this review may have occurred through genotype differences in BMI, but the majority of the effect is likely to be due to independent effects of alcohol on blood pressure.

It is possible that the ALDH *2*2 genotype influenced blood pressure directly or by another mechanism that was independent of the effect on alcohol intake (pleiotropy). Analyses of this genotype in relation to oesophageal cancer are complicated by dual effects of the ALDH2 *2*2 genotype in reducing alcohol intake but dramatically increasing acetaldehyde, a potent carcinogen, among those who do drink [9]. Elevation of acetaldehyde causes vasodilation, and consequently lower blood pressure, which is associated with increased skin temperature and characteristic flushing observed among ALDH2 *2*2 homozygotes [27]. The lower blood pressure associated with acetaldehyde could account for the acute reduction in blood pressure immediately after ingesting alcohol. However, this effect is short lived, and acetaldehyde is unlikely to explain the lower blood pressure seen among *2*2 homozygotes in this study, particularly as they tend not to drink. In fact, heterozygotes tend to have higher levels of acetaldehyde than either *1*1 or *2*2 homozygotes, because although they drink alcohol in moderation, they clear acetaldehyde at a much slower rate and consequently have greater elevations of acetaldehyde per unit of alcohol consumed than do *1*1 homozygotes, which is illustrated by their high oesophageal cancer risk relative to other genotypes [9]. Therefore if the association between genotype and blood pressure were due to acetaldehyde levels rather than alcohol intake per se we would expect heterozygote individuals to have the lowest blood pressure and the lowest risk of hypertension. It is also possible that these results have arisen by confounding due to close proximity of the genotype of interest with other genotypes that may influence blood pressure (the close proximity of genotypes at different loci such that they are inherited together is termed linkage disequilibrium). Evidence that the effects of this genotype on blood pressure are due to differences in alcohol consumption levels rather than pleiotropic effects of *ALDH2* or linkage disequilibrium, is provided by the data on women, who tended not to drink and among whom there were no differences in blood pressure by genotype. If either pleiotropic effects or confounding by linkage disequilibrium were operating, an effect on blood pressure would be seen in both sexes.

Additional evidence that argues against linkage disequilibrium and pleiotropic effects and an effect of acetaldehyde on blood pressure, is that among studies that looked at the effect of *ALDH2* genotype after adjustment or stratification for amount of alcohol consumed there was no difference in blood pressure by genotype [11,13,14,18,20,28,29]. The exception is a study that found a residual association between *ALDH2* and blood pressure in a multiple logistic regression analysis including age, BMI, genotype, and alcohol consumption, but the association with genotype in this analysis was considerably weaker than in the univariate analysis, and the authors concluded that this difference could be due to differences in past drinking habits between genotype groups, which were not controlled for in the analysis [19]. The finding could also be due to measurement error in self-reported alcohol intake data or chance.

There was some evidence of small-study effects in our review, which suggests that publication bias may have occurred. This bias may have led to an overestimation of the effects in the meta-analyses. However, only three of the largest studies were included in our instrumental variable analysis, as these were the only studies that presented mean alcohol intake by genotype, and so we do not expect this estimate to be influenced to a great extent by small-study bias; in addition a sensitivity analysis including only studies with at least 500 participants showed only minor changes in effect estimates.

In summary, observational epidemiological studies have proved to be a misleading source of knowledge regarding the cardioprotective effects of several dietary factors [30]. Inadequate statistical control for confounding by other behavioural and sociodemographic factors related to cardiovascular disease risk are likely to underlie this problem. This study shows that alcohol intake may increase blood pressure to a much greater extent, even among moderate drinkers, than previously thought. Large-scale replication studies are required to confirm this finding and to improve the precision of our estimates.

## Supporting Information

Alternative Language Abstract S1 Translation of the Abstract in Chinese by Lina Chen. (20 KB DOC)

## Abbreviations

BMI: body mass index
CI: confidence interval
DBP: diastolic blood pressure
SBP: systolic blood pressure
