# Supplementary material for: Alcohol Intake and Blood Pressure: A Systematic Review Implementing a Mendelian Randomization Approach
Source: PLoS Med. 2008 Mar 4;5(3):e52. doi: 10.1371/journal.pmed.0050052 (PMC2265305; doi:10.1371/journal.pmed.0050052)
Supplement: Supplementary file 1 [file pmed.0050052.sd001.doc]

摘要：

**背景材料**：饮酒被证明是常见的并可调节的导致高血压疾病的危险因素。但是，目前关于饮酒和高血压症的观察研究结果经常被一些其它因素如：个人行为和社会地位等所干扰，而相关的临床实验则难以落实并且跟踪时间有限。根据孟德尔随机定律，*aldehyde dehydrogenase 2* (*ALDH2*)基因上的常见的遗传多样性可以作为酒精摄取量的测量尺度，从而为研究饮酒和高血压症的关系提供切实可靠的证据。*ALDH2*的蛋白产物是主要的酒精代谢酶之一。携带其纯合突变型的人 (**2*2*) 在饮酒过程中会产生不适症状，因此他们的饮酒量要相对少于野生型 (**1*1*) 或杂合型携带者。据此，我们假设这个遗传多样性可以通过干扰酒精摄取量从而影响高血压症的得病风险。

**方法与结果**：我们应用系统回顾和固定效应的综合分析方法研究*ALDH2*基因型和血压值（3个论文，总人数=4219）以及和高血压症的关系（5个论文，总人数=7658）。在男性中，我们通过比较获得**1*1* 野生型携带者整体高血压症致病几率是**2*2*突变型携带者的2·42 倍(odds ratio) (95% CI: 1·66-3·55, p=4·8×10^-6^)；而杂合型携带者（中度饮酒者）致病几率则是**2*2* 突变型携带者的1·72倍 (95% CI: 1·17-2·52, p=0·006)。野生型**1*1*携带者的收缩压高于突变型**2*2* 携带者 7·44 mmHg (95% CI: 5·39-9·49, p=1·1×10^-12^) ，而杂合型携带者的收缩压则高出 4·24mmHg (95%CI: 2·18-6·31, p=0·00005)。

**结论**：以上结果支持了饮酒可以影响血压并可能导致高血压症的假设。
